# Efficacy of Anti-Interleukin-2 Receptor Antibody (Daclizumab) in Reducing the Incidence of Acute Rejection After Renal Transplantation

**DOI:** 10.5812/numonthly.1806

**Published:** 2012-03-01

**Authors:** Hossein Saghafi, Khosrow Rahbar, Ali Nobakht Haghighi, Mohammad Qoreishi, Farshad Safdari

**Affiliations:** 1 Department of Nephrology, School of Medicine, Qom University of Medical Sciences, Qom, IR Iran; 2 Department of Nephrology, School of Medicine, Shahid Beheshty University of Medical Science, Tehran, IR Iran; 3 Department of Orthopedic Surgery, School of Medicine, Shahid Beheshty University of Medical Sciences, Tehran, IR Iran; 4 Akhtar orthopedic Research Center, Shahid Beheshty University of Medical Sciences, Tehran, IR Iran

**Keywords:** Daclizumab, Graft Rejection, Renal Transplantation

## Abstract

**Background:**

Acute rejection remains a major problem in renal transplantation and represents one of the most important causes of chronic allograft dysfunction and late graft loss. Daclizumab is a genetically engineered human IgG1 monoclonal antibody that binds specifically to the α chain of the interleukin-2 receptor, and may thus reduce the risk of rejection after renal transplantation.

**Objectives:**

The aim of this study was to examine the effect of daclizumab induction therapy combined with a triple immunosuppressive protocol including prednisolone,cyclosporine microemulsion (CsA), and mycophenolate mofetil (MMF), in reducing the incidence of acute rejection in recipients of living unrelated donor kidneys.

**Patients and Methods:**

In this historical cohort study, 43 adult recipients of their first kidney allograft received daclizumab (three 1 mg/kg doses administered every 2 weeks) with triple immunosuppressive therapy (steroids, CsA, and MMF). This group was compared to 43 first-time graft recipients who received maintenance triple immunosuppressive therapy comprising steroids, CsA, and MMF. The end point was the incidence of biopsy confirmed acute rejection within 6 months after transplantation.

**Results:**

At 6 months, 5 (11.6%) of the patients in the daclizumab group had biopsy-proven rejections, as compared to 14 (32.5%) in the control group (P = 0.017). The sex and the age of recipients had no impact on the incidence of acute rejection episodes in the two groups.

**Conclusions:**

Adding interleukin-2 receptor antibody (daclizumab) to maintenance triple immunosuppressive therapy (prednisolone, CsA, and MMF) reduces the incidence of acute rejection episodes at 6 months in first-time transplant recipients of living unrelated donor.

## 1. Background

The survival of renal allograft transplantation, which is the treatment of choice for patients suffering from end-stage renal disease, has increased in recent decades (1). This is primarily attributable to the development of new immunosuppressive drugs (1). One of the important predictors of allograft survival is the incidence of acute rejection (AR) during the first 3–6 months postoperatively (1, 2). The more frequently AR occurs, the higher the possibility that early renal allograft dysfunction will occur subsequently (2). Previously, this problem affected 20%–50% of transplantations and caused renal allograft loss in 5%–10% of the patients (3). Despite recent advances, AR episodes represent important challenges during the postoperative care of kidney transplant recipients (3).

## 2. Objectives

Several studies have shown that interleukin 2 receptor antibody (daclizumab) can reduce acute rejection episodes in high-risk kidney transplant recipients. In the current study, we proposed to evaluate the efficacy of daclizumab in low-risk first-time transplant recipients of living unrelated donor kidneys.

## 3. Patients and Methods

In this historical cohort study, 86 patients who underwent their first live renal transplantation were examined in the early period after transplantation. This survey was carried out between 2004 and 2008 in Taleghani Hospital, Tehran. All participants gave verbal informed consent following an explanation of the procedures involved in the study. Consent to perform the study was obtained from our hospital ethics committee.

The baseline maintenance immunosuppressive therapy protocol including prednisolone, cyclosporine microemulsion (CsA = 5 mg/kg/d), and mycophenolate mofetil (MMF = 2 g/d) was administered to all patients. Subjects were divided into two groups of the same size. The case group had 27 men and 16 women and the control group had 25 men and 18 women. Patients in the case group received 1 mg/kg daclizumab (Zenapax; Roche, Basel, Switzerland) every 2 weeks for three doses in addition to the baseline maintenance therapy. The two groups were matched with age, sex of donors and recipients, the cause of end-stage renal disease, and the cytomegalovirus and Epstein-bar virus serologic status of donors and recipients. All patients were followed for 6 months. Exclusion criteria included age less than 18 years, HIV, hepatitis B infection, or active and significant infectious conditions. In addition, participants were excluded if they were receiving therapy with other immunosuppressive regimens. In patients presenting with renal dysfunction, we initially ruled out other causes such as renal artery stenosis, viral and other infectious diseases, cyclosporine overdose, or urinary tract obstruction. Acute rejection was diagnosed based on clinical signs and symptoms, physical examination, and histological confirmation from a renal biopsy by an expert pathologist. The results of histological studies were positive based on the presence of acute tubulitis or vasculitis. Finally, the data were analyzed using the chi-square test. Results were considered statistically significant if the P-value was <0.05.

## 4. Results

**Figure 1:**
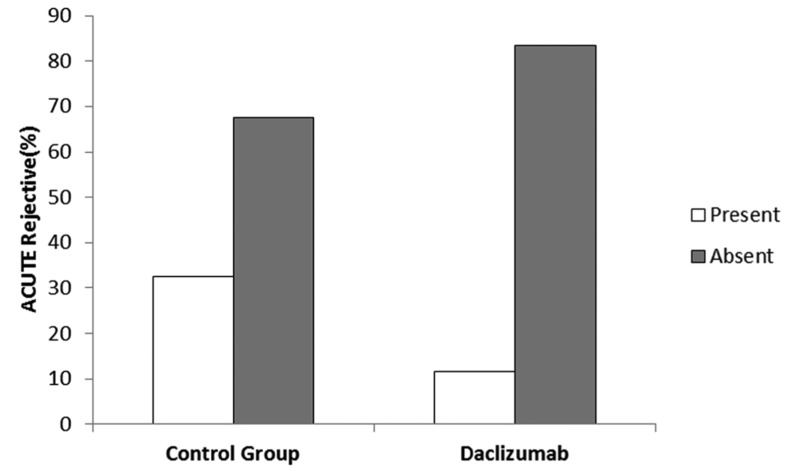
The Incidence (percentage) of Acute Rejection in the Control and Daclizumab Groups Within 6 Months After Transplantation.

There were 27 men and 16 women in the case group (daclizumab), with a mean age of 35.9 ± 8.1 years. The mean age in the control group, comprising 25 men and 18 womenwas 35.7 ± 8.2 years. We found that AR, as defined by the histological analysis of a biopsy sample, occurred in 19 (22%) patients during the 6-month postoperative period. Five (11.62%) of these patients were in the daclizumab group (2 women and 3 men) and the remaining 14 (32.5%) were in the control group (5 women and 9 men) (Figure 1). There was a statistically significant difference between the groups (P = 0.017). According to the current study, age and gender did not affect the incidence of AR.

## 5. Discussion

Renal allograft transplantation is the treatment of choice for patients with ESRD. Episodes of acute rejection are an important predictor of renal function and graft loss in renal allograft recipients. Recently, because of exact and detailed preoperative laboratory assessments and newer immunosuppressive drugs, the incidence of AR has decreased.Even though appropriate treatments help the management of the patients with AR, it should be remembered that this problem may decreaseallograft survival by 50% (2, 4). Daclizumab is a recombinant humanized immunoglobulin G1 subclass monoclonal antibody that specifically blocks the α subunit (CD 25) of interleukin-2 (IL-2) receptor, which is expressed on the surface of activated lymphocytes (5-8).

The use of antibody induction after kidney transplantation has increased from 25% to 63% in the past decade. The induction agent used in approximately half of the patients is IL-2RA, i.e., basiliximab or daclizumab (9). Several studies have demonstrated the benefits of daclizumab in high-risk renal allograft transplantation from deceased donors or in kidney recipients with positive WBC cross-match (2, 8-11). In the current study, we showed that daclizumab is effective in reducing the incidence of acute rejection in first-time kidney transplant recipients from unrelated live donors, if administered together with baseline maintenance immunosuppressive therapy, including prednisolone, cyclosporine microemulsion, and mycophenolate mofetil as an induction therapy. Bumgardner et al. (12) found that daclizumab decreases the incidence of biopsy-proven AR at 1 year post-transplantation, and Ekberg et al. (13), Meier-Kriesche et al. (14), Millan et al. (15), and Morris et al. (16) obtained similar results in their studies. Kandus (17) showed that basiliximab or daclizumab combined with triple therapy was an efficient and safe immunosuppressive strategy, which was demonstrated by the low incidence of acute rejections, excellent graft function, high survival rates, and an acceptable adverse event profile in adult recipients within the first year after renal transplantation with kidneys from deceased donors.

Daclizumab combined with triple immunosuppressive therapy, including steroids, CsA, and MMF, reduces the incidence of AR in low-risk firsttime recipients of transplanted kidneys from unrelated living donors. Further studies are needed to evaluate the overall benefits of these new strategies on the long-term survival of patients and allografts.
